# Paratonia is inversely correlated to premotor areas perfusion

**DOI:** 10.1097/WNR.0000000000002317

**Published:** 2026-11-11

**Authors:** Lucio Marinelli, Daniele Diotti, Luca Puce, Antonio Currà, Nicola Girtler, Matteo Pardini, Stefano Raffa, Gianmario Sambuceti, Silvia Morbelli, Carlo Trompetto

**Affiliations:** a Department of Neuroscience, Rehabilitation, Ophthalmology, Genetics, Maternal and Child Health, University of Genoa; b AOM - IRCCS Ospedale Policlinico San Martino, Genova; c CRIAMS-Sport Medicine Centre Voghera, University of Pavia, Voghera; d Department of Medico-Surgical Sciences and Biotechnologies, DAI Neurosciences and Mental Health, AOU Policlinico Umberto I, Sapienza University of Rome, Rome; e Department of Health Sciences, University of Genoa, Genova; f Department of Medical Sciences, University of Turin; g Nuclear Medicine Unit, Azienda Ospedaliero-Universitaria Città della Salute e della Scienza di Torino, Turin, Italy

**Keywords:** Alzheimer’s disease, frontal lobe, long-loop reflexes, motor output, muscle hypertonia, muscle relaxation, response inhibition

## Abstract

**Objective:**

Paratonia is defined as an inability to relax the muscles during tone assessment, resulting in participants either opposing or facilitating the examiner’s movement involuntarily. This phenomenon is frequently found in patients with cognitive impairment and has been attributed to frontal lobe dysfunction, although a direct correlation has not been established. This study aimed to explore the correlation between frontal lobe function, as assessed by functional brain imaging, and paratonia, as assessed by electromyography.

**Methods:**

Memory clinic outpatients with suspected Alzheimer’s disease who were prescribed an amyloid PET scan underwent a thorough neuropsychological examination and a paratonia assessment involving surface electromyography recording of the elbow flexors and extensors. PET image acquisition started immediately after tracer injection, and the early phase perfusion amyloid PET was used as a surrogate biomarker of neuronal injury. Multiple regression analysis was performed on amyloid-positive patients to identify the correlation between paratonia and brain perfusion.

**Results:**

Twenty-one amyloid PET-positive participants with a median age of 72 years were diagnosed with Alzheimer’s disease and included in the analysis. All participants were right-handed, and paratonia was assessed in the left arm. Whole-brain voxel-wise analysis revealed a significant inverse correlation between the total paratonia score and cortical perfusion in a cluster encompassing the superior frontal gyrus in the left hemisphere (Brodmann areas 6, 8, and 9; *P* < 0.006).

**Conclusion:**

This study demonstrates for the first time an inverse correlation between prefrontal perfusion and paratonia. Participants with better-preserved premotor function also show less paratonia, as reflected by reduced electromyographic activation of the muscles undergoing passive mobilization.

## Introduction

Paratonia has been defined as the inability to relax muscles during muscle tone assessment, with oppositional paratonia offering resistance to passive movements and facilitatory paratonia occurring when the action is in the same direction as passive movement. Both types of paratonia have been associated with cognitive impairment or mental disorders [1–6]. Studies suggest that, among primitive reflexes, (oppositional) paratonia is the one showing the highest negative correlation with neuropsychological tests (registration, orientation, verbal fluency, recall, drawing) [3]. Nevertheless, paratonia can be clinically detected in a minority (5%) of participants without cognitive impairment [3,7]. We developed a method based on surface electromyography able to detect slight paratonic muscle activation even in participants where traditional approaches do not allow the detection of paratonia [8]. This approach allows a more sensitive and quantitative measure of paratonia. EMG activity during upper limb passive mobilization resembling paratonia can be detected even in participants without cognitive impairment and without clinically detectable paratonia. Indeed, the amplitude of paratonic EMG bursts was higher in participants with paratonia and lower in those without, though it was also detectable in the majority of young, healthy participants. It showed the same features as in participants with cognitive impairment, namely an increase with velocity and movement repetition [9]. The presence of paratonia has been attributed to frontal lobe dysfunction for many years [10] but the correlation between frontal lobe function and paratonia was demonstrated only with a neuropsychological approach [2]. The presence of paratonia also in healthy participants is not unexpected and can be attributed to a paraphysiological reduced inhibitory efficiency of frontal areas [11]. Interestingly, this variable inhibitory efficiency of frontal areas can be measured thanks to surface electromyography [9]. We recently demonstrated that, in young, healthy participants, the amount of EMG-recorded paratonia depends on their level of physical activity. Sedentary participants show higher paratonia than athletes, and this difference is even more pronounced when the sporting activity involves the same limb that is being assessed for paratonia. This suggests that the efficiency with which frontal areas inhibit movement can probably be trained [12].

Brain amyloidosis is the signature of Alzheimer’s disease pathology and is detectable at various degrees in Alzheimer’s disease, dementia with Lewy bodies, and in the apparently healthy elderly population as well. Recently, amyloid PET studies have made it possible to detect brain amyloidosis *in vivo* and to evaluate its regional distribution. Amyloid PET is commonly used as a diagnostic tool to assess the presence of Alzheimer’s disease pathology *in vivo*, via assessment of images acquired 90 min after radiotracer administration. However, new approaches based on acquiring images within minutes of radiotracer administration (i.e. early-phase amyloid PET imaging) can capture blood flow distribution and thus provide an estimate of relative cerebral blood flow, and indirectly of regional neurodegeneration [13]. In fact, acquired immediately after tracer injection, early-phase PET perfusion maps mirror regional cerebral blood flow and have been demonstrated to correlate with glucose metabolism measured by fluorodeoxyglucose (FDG) PET [14,15]. Neurodegenerative disorders are characterized by the loss of neurons and reduced metabolism in the affected areas. This single-tracer, dual-phase protocol allows clinicians to obtain both a perfusion-based surrogate of neurodegeneration and a late-phase metric of amyloid plaque load within a single session, potentially minimizing costs and patient burden [14]. According to appropriate criteria of clinical use [16], an amyloid PET scan is indicated for those patients with suspected Alzheimer’s disease but who remained undiagnosed after an extensive first- and second-level investigation panel. Showing a negative amyloid PET scan in those participants virtually excludes Alzheimer’s disease, while a positive examination stands for Alzheimer’s disease only if it has been performed for confirmation purposes in a patient with clinically suspected Alzheimer’s disease. Moreover, both amyloid PET-negative and positive patients show different degrees of amyloid load along a continuum ranking from the most negative to the most positive ones.

We aim to further explore the correlation between frontal lobe functioning and EMG-assessed paratonia by regional cerebral blood flow (rCBF) in amyloid-positive participants and possibly define the involved frontal areas and hemispheric prevalence. Specifically, we hypothesize that participants with more severe paratonia will also show less frontal inhibitory efficiency, as reflected by reduced rCBF in frontal and likely premotor areas.

## Materials and methods

### Participants

Outpatients from the Memory Clinic at the ‘San Martino’ Hospital, IRCCS Azienda Ospedaliera Metropolitana (Genova, Italy) who were prescribed an amyloid PET scan were asked to undergo EMG recording of paratonia. Demographic data and clinical characteristics of the enrolled participants, as well as their clinical diagnosis, neuropsychological examination, and neuroimaging findings (brain MRI), have been collected.

Exclusion criteria are the following: (a) presence of Parkinsonian features, (b) history of stroke or other acute brain lesions, and (c) inability to provide informed consent.

All patients underwent an extended neuropsychological battery tailored to unveil mild signs of cognitive impairment in the domains of language, visuoconstruction, attention, executive functions, and memory. The drug regimen was also carefully collected. Facilitatory and oppositional paratonia was assessed in all participants using the paratonia scale and the modified Kral procedure [2] before the electromyographic recording.

All participants gave informed consent to all study procedures, which were performed according to the Declaration of Helsinki. This observational study was approved by the ‘Comitato Etico Territoriale - Liguria’ ethics committee (approval number 15/2025 – id 14261).

### Movement evaluation with electromyographic recordings

All participants were seated in a comfortable armchair. The examiner was seated near the patient, holding one of the patient’s arms (nondominant side), and listening to the tones produced by a software-emulated metronome through earphones. Surface preamplified electrodes with fixed inter-electrode distance (TSD150B; Biopac Systems Inc., Goleta, California, USA) were placed over the muscle belly of the biceps and triceps brachii following current guidelines [17]. The distance between the two preamplified electrodes was maximized to reduce cross-talk. The excursion of the elbow joint was continuously recorded by a twin-axis electronic goniometer placed across the joint (TSD130B; Biopac Systems Inc.). All signals were acquired by a Biopac MP150 unit (Biopac Systems Inc.) for later offline analysis.

Continuous and discontinuous elbow movements with reproducible angular velocity were obtained following a previously described method [18]. During the procedure, participants were instructed to remain relaxed and to avoid resisting or facilitating the movements performed by the examiner. A baseline recording at rest confirmed the absence of electromyographic activity in both biceps and triceps muscles. Movement velocity and frequency were determined by the metronome [e.g. 60 beats per minute (BPM) – generating tones at a 1 s interval]. Following the rhythm, the examiner moved the patient’s forearm resulting in consecutive, smooth flexion-extension of the forearm, reaching the maximal flexion and extension in synchrony with the metronome tones. For each patient, we recorded 60 flexion and 60 extension cycles, including faster (100 BPM), slower (60 BPM), continuous, and discontinuous movements performed in a random order. The electromyographic activity resulting from maximal voluntary contraction was also recorded from both biceps and triceps. The electromyographic recording of paratonia was performed on the left arm just before PET/computed tomography (CT) image acquisition.

### PET/computed tomography image acquisition

Amyloid-PET images were acquired using an ^18^F-flutemetamol tracer according to European Association of Nuclear Medicine/Society of Nuclear Medicine and Molecular Imaging joint guidelines [19]. Briefly, late standard amy-PET images were acquired 90 min after the intravenous administration of 185 MBq (4 × 5-min image frames). Images were then averaged into a single 20-min frame. All amyloid-PET late images were visually assessed by a Nuclear Medicine specialist certified for amy-PET reading (S.M.) applying the standard operating procedures approved by the European Medicines Agency. Only patients with a positive amyloid PET were included in the statistical parametric mapping (SPM) analysis.

Early-phase amyloid-PET was used as a surrogate biomarker of neuronal injury. Image acquisition was started immediately after tracer injection (via venous cannula), and a static image was acquired for 6 min. Current amyloid-PET imaging guidelines mention the possibility of performing early perfusion imaging without detailing the acquisition procedure or its specific clinical utility. Early acquisition within 5–10 min postinjection represents the most common protocol among selected studies, as highlighted in a recent European Association of Nuclear Medicine perspective on early-phase imaging [14]. Within this framework, we adopted the slightly shorter acquisition window, which appears to correlate more closely with R_1_ and ^18^FDG PET [20–23]. This choice is particularly relevant for positive (late-phase) amyloid-PET scans – such as those in the present study – where reduced early tracer washout can potentially mask regional hypoperfusion. Data were acquired in list mode and reconstructed using three-dimensional (3D) Fourier rebinning-ordered subsets expectation-maximization interactions, eight subsets, and a 2 mm Gaussian filter at full width and half maximum (FWHM).

### EMG data analysis

After filtering the EMG signal using notch and bandpass filters (20–250 Hz) and rectifying it, the recordings were visually inspected offline to exclude artifacts. For each patient, the ‘mean_EMG’ value, which reflects the average amplitude of the EMG signal during each movement and is normalized by the maximal voluntary contraction, was calculated using the ‘mean’ function of the AcqKnowledge software (version 4.2; Biopac Systems Inc.) [8,18]. A pure number reflecting EMG activity was obtained for each passive movement. Then, for each subject, the average value, which we here call the ‘total paratonia score’, was calculated.

### PET/computed tomography image analysis

Processing was performed using stand-alone SPM (SPM12; Wellcome Trust Centre for Neuroimaging, London, UK) [24]. Early perfusion amyloid PET images were normalized to the Montreal Neurological Institute space and then spatially smoothed with an isotropic 3D 8 mm Gaussian kernel (FWHM).

After preprocessing, multiple regression analysis in SPM12 was used to identify the correlation between total paratonia score and brain perfusion. Age, gender, and mini-mental status examination (MMSE) were included as nuisance variables. To account for intersubject variability in MMSE scores, the analyses were repeated by including the MMSE score as an additional nuisance covariate in 20 of the 21 patients (as one MMSE score was unavailable). An family-wise error-corrected height threshold less than 0.05 at both peak and cluster levels was considered significant. Only clusters with size *k* ≥ 100 voxels were considered.

## Results

Twenty-one amyloid-PET-positive participants, nine males, with a median age of 72 years [range: 55–79, interquartile range (IQR): 66–78] were included in the analysis. All participants were diagnosed with Alzheimer’s disease based on the clinical history, cognitive profile, and amyloid PET results, with a median MMSE score of 25 (range: 14–28, IQR: 16–26), although the test could not be performed in one subject (#20) because of prevalent speech production impairment. The participants presented with a negative brain MRI study for space-occupying lesions and for severe chronic ischemic vascular load. All participants were right-handed, so paratonia was always assessed in the left arm. Whereas paratonia was clinically detected in 19 (90%) participants, EMG activity reflecting paratonic activation was recognizable in 100% of the participants, at least during some of the passive movements (Table 1).

**Table 1:** Recruited participants

Subject, *n*	Age	Gender	MMSE	FacPS	OppPS	Kral	TPS
1	55	F	16	2	1	0	23.3
2	75	F	26	1	0	0	15.5
3	78	F	28	1	1	0	61.0
4	71	M	14	2	0	0	12.1
5	68	M	28	2	1	2	28.5
6	75	F	23	2	2	2	55.9
7	79	F	14	3	3	2	78.7
8	57	M	25	2	1	1	11.1
9	77	F	25	2	3	2	6.7
10	79	M	26	2	1	1	70.8
11	78	F	27	2	2	0	132.5
12	63	M	26	2	2	1	28.9
13	75	F	16	0	0	0	14.9
14	64	M	25	0	0	0	0.6
15	71	M	25	2	1	1	6.2
16	62	F	17	1	2	0	48.9
17	67	M	15	2	1	0	240.0
18	78	F	24	3	0	2	89.1
19	67	M	25	2	1	2	14.5
20	72	F	n/a	3	1	2	54.2
21	77	F	26	4	1	2	70,0

FacPS, facilitatory score for paratonia scale; Kral, score for modified Kral Procedure; MMSE, mini-mental status examination; OppPS, oppositional score for paratonia scale.

Electromyographic recording of paratonia was performed on the left arm right before PET/CT image acquisition.

Whole brain voxel-wise analysis revealed a significant inverse correlation between total paratonia score and cortical perfusion in a cluster encompassing the superior frontal gyrus in the left hemisphere (Brodmann areas 6, 8, and 9; *P* < 0.006); the inclusion of MMSE as a further nuisance variable in 20 out of 21 patients did not change the results (Fig. 1). No cortical regions showed a direct correlation with quantitative measures of paratonia.

**Fig. 1:**
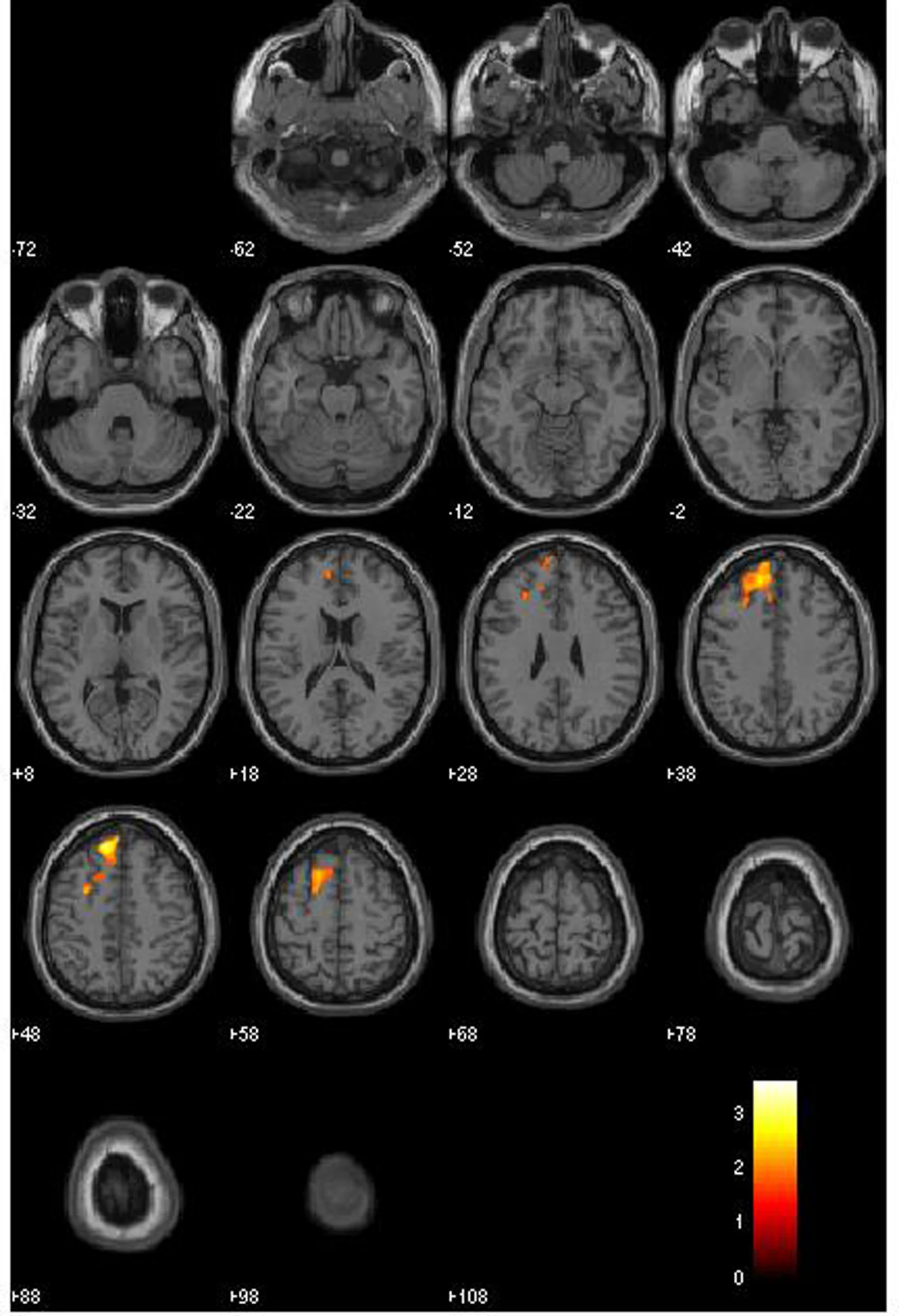
Correlation between brain perfusion as measured with early frame amyloid PET and paratonia score. Whole-brain voxel-wise analysis revealed a significant inverse correlation between total paratonia score and cortical perfusion in a cluster encompassing the superior frontal gyrus in the left hemisphere (Brodmann areas 6, 8, and 9; *P* < 0.006). Clusters with significant correlation are shown superimposed on an MRI template [peak MNI coordinates: −12, 44, 42 (*T*: 3.51; *r*: −0.66); cluster size: 246, voxel-wise *P* < 0.001, and cluster *P* < 0.006]. The color bar indicates the level of *Z* scores for significant voxels (yellow: highest; red: lowest). MNI, Montreal Neurological Institute.

## Discussion

The conjoint acquisition of EMG-recorded paratonia in the left upper limb and functional brain imaging in 21 amyloid-positive participants demonstrates an association between EMG-assessed paratonia and early-phase PET signal in left superior frontal regions. Participants with a higher perfusion of the superior frontal gyrus in the left hemisphere (Brodmann areas 6, 8, and 9) also had less paratonia in the biceps brachii and triceps brachii of the left upper limb, therefore supporting an inverse correlation between left premotor areas’ perfusion and paratonia. This inverse correlation is independent of age, gender, and cognitive status, as assessed using the MMSE. Our instrumental finding confirms previous findings obtained by evaluating paratonia with clinical scales (paratonia scale and modified Kral procedure) and frontal lobe function with neuropsychological assessments (Luria’s tests for echopraxis and the controlled word association test) [2]. The frontal region showing a significant inverse correlation with paratonia is rather large, encompassing not only the premotor cortex and supplementary motor area (SMA, Brodmann area 6), but also frontal eye fields (FEFs, Brodmann area 8) and dorsolateral prefrontal cortex (DLPFC, Brodmann area 9).

The involvement of the premotor cortex and particularly the SMA in the generation of paratonia is in agreement with the inhibitory role of the premotor cortex. According to an enlightening fMRI study, SMA activation is larger during muscle relaxation than contraction [25]. It should be noted that the motor tasks performed in the study always involved the distal part of the right upper arm (pressing the right thumb against the index and middle fingers, and extending the wrist), but SMA activation was bilateral. Preparation of volitional movements in humans is associated with a negative scalp-recorded potential called the Bereitschaftspotential, which is considered a neurophysiological correlate of SMA activation. Interestingly, this potential is generated not only during motor preparation, but also during voluntary muscle relaxation or voluntary termination of muscle contraction [26]. The correlation between paratonia and Brodmann areas 8 and 9 is consistent with evidence suggesting that FEF and DLPFC play a role in response inhibition, although with respect to the inhibition of ocular saccades rather than limb movements [27]. The DLPFC’s role in working memory and attention has been demonstrated, and, along with FEF and pre-SMA, it is involved in stimulus selection and response inhibition in go/no-go tasks [28]. Considering that perfusion in these areas was not assessed online during passive mobilization, but at rest, it is likely that the correlation with paratonia is not strictly related to the motor task. Patients with higher SMA perfusion are also preserved in immediately adjacent areas such as the FEF and DLPFC and therefore, in addition to having less paratonia, are likely to perform better in tasks involving motor response inhibition. In fact, a recent study has shown that athletes, as healthy participants with fine motor control, have less paratonia than sedentary participants [12].

In this study, passive mobilization for EMG recording of paratonia was performed only in the upper left (nondominant) arm, and the correlation with prefrontal perfusion was found only with the left side. Although this correlation only with ipsilateral areas was partly unexpected, it must be noted that premotor areas’ role in motor inhibition is not necessarily dependent on the assessed body side. In a study assessing premotor activity using functional near-infrared spectroscopy, in right-handed participants, the left premotor cortex played a major role in muscle relaxation even when hand movements were performed with the left ipsilateral hand [29]. Moreover, it has been demonstrated that strong ipsilateral activity in the left motor cortex is specific to complex movements independently of the number of required muscles, indicating a prominent role of the left hemisphere [30]. As all participants in this study were right-handed, it is possible that the correlation found in the left hemisphere may be due to hemispheric dominance. Studies involving left-handed participants or those correlating paratonia scores between the two sides of the body could probably help to clarify these aspects.

A unifying pathophysiological hypothesis to explain how paratonia is generated and why it increases when frontal (premotor) efficiency is reduced consists of a sensory-motor long loop involving the muscles undergoing passive mobilization and the inhibitory role of the SMA on more caudal areas responsible for paratonia generation. During passive limb mobilization, it is conceivable that the repeated activation of muscle spindles activates afferent sensory pathways that reach the primary sensory cortex. This then activates the primary motor cortex, producing a descending volley back to the mobilized muscles in either a facilitatory or oppositional way. The primary motor cortex is actively and voluntarily inhibited by the SMA [25], with the likely involvement of contiguous premotor areas that could engage with rhythmic events (Fig. 2). Weaker SMA inhibitory control over posterior motor areas could explain why paratonia persists in participants with defective frontal lobe function due to neurodegenerative conditions or focal lesions, as well as in healthy participants with less experience controlling fine movements. Conversely, stronger SMA activity in young and healthy athletes may justify their much lower or absent paratonic activity [12]. Further studies are needed to confirm this hypothesis.

**Fig. 2:**
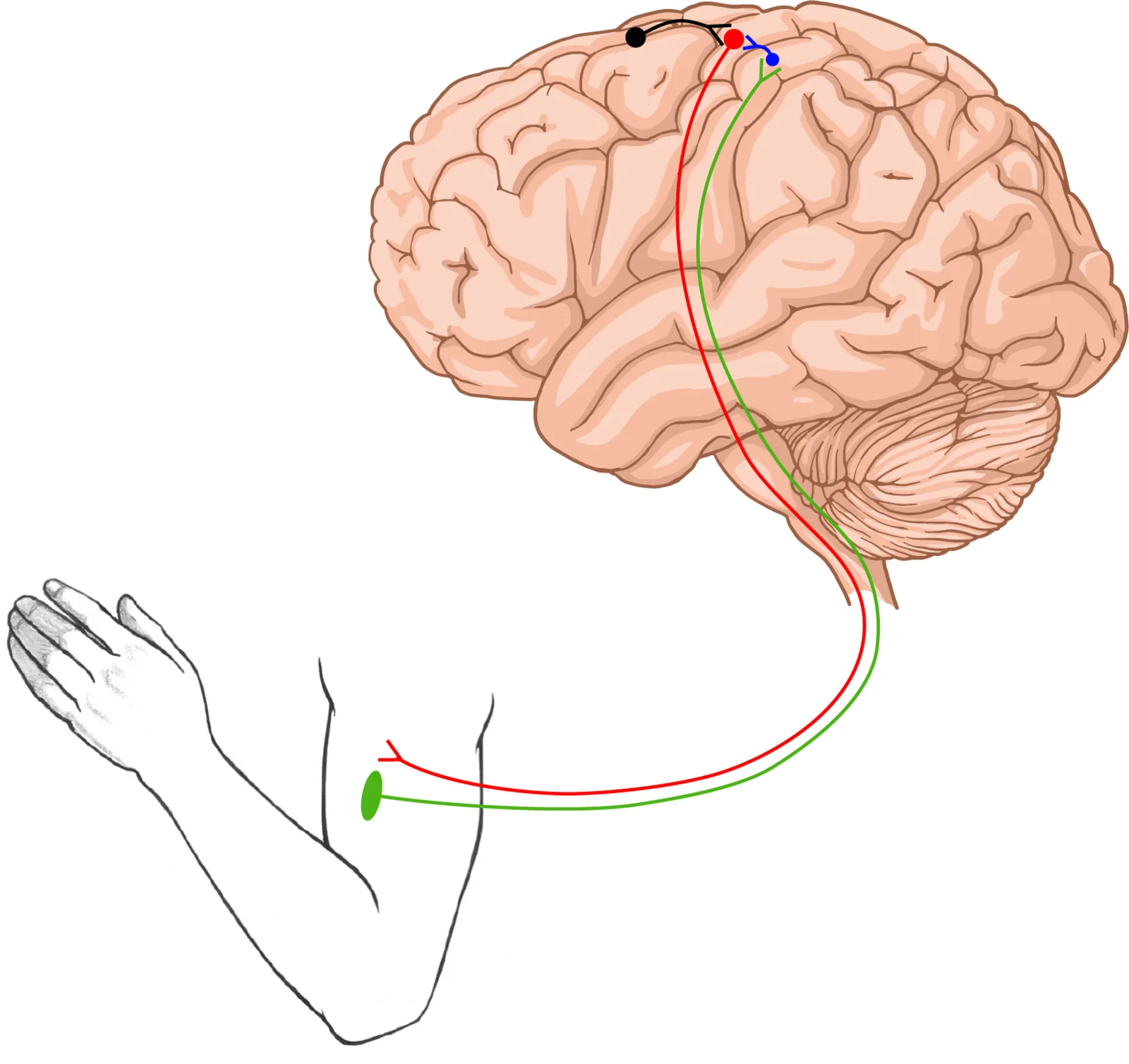
Hypothetical long-loop network subtending paratonia. During passive elbow mobilization, the alternate shortening and stretching of the flexor and extensor muscles activates proprioceptive sensory pathways that ultimately reach the primary somatosensory areas (green) and then the motor areas (blue). This activates the descending motor pathways (red). However, this reflex motor output is actively inhibited by the premotor areas (black), particularly the supplementary motor area.

We acknowledge several limitations: the limited number of recruited participants, the lack of differentiation between muscles (biceps and triceps brachii) and paratonia type (facilitatory and oppositional), the fact that early-phase amyloid PET is only an indirect measure of regional brain function, and also the lack of a control group because of the infeasibility of performing CT/PET scans in normal participants. Finally, despite MMSE being included as a nuisance variable in the SPM analysis, we cannot exclude an impact of the relatively wide range of cognitive severity in the present study.

### Conclusion

Paratonia is not only related to cognitive decline but is also age-related and generally reflects reduced strength or disruption of prefrontal inhibitory circuits. Specifically, if recorded with EMG, paratonia could be explored in subsequent studies centered on premotor inhibition or, more broadly, frontal inhibitory function, during longitudinal evaluations and to examine the impact of pharmacological therapies or rehabilitation.

## Acknowledgements

This work was supported by the Italian Ministry of Research, under the complementary actions to the NRRP ‘Fit4MedRob - Fit for Medical Robotics’ Grant (# PNC0000007).

### Conflicts of interest

There are no conflicts of interest.
